# Stepwise differentiation of functional pancreatic β cells from human pluripotent stem cells

**DOI:** 10.1186/s13619-022-00125-8

**Published:** 2022-08-01

**Authors:** Wenwen Jin, Wei Jiang

**Affiliations:** 1 Department of Biological Repositories, Frontier Science Center for Immunology and Metabolism, Medical Research Institute, Zhongnan Hospital of Wuhan University, Wuhan, 430071 China; 2 Human Genetics Resource Preservation Center of Wuhan University, Wuhan, 430071 China

**Keywords:** Pancreatic β cell, Human pluripotent stem cells, Stepwise differentiation, Diabetes mellitus

## Abstract

Pancreatic β cells differentiated from stem cells provide promise for cell replacement therapy of diabetes. Human pluripotent stem cells could be differentiated into definitive endoderm, followed by pancreatic progenitors, and then subjected to endocrinal differentiation and maturation in a stepwise fashion. Many achievements have been made in making pancreatic β cells from human pluripotent stem cells in last two decades, and a couple of phase I/II clinical trials have just been initiated. Here, we overview the major progresses in differentiating pancreatic β cells from human pluripotent stem cells with the focus on recent technical advances in each differentiation stage, and briefly discuss the current limitations as well.

## Background

Diabetes mellitus, characterized by severe hyperglycemia, has been estimated to affect 537 million people worldwide and over 140 million Chinese people in 2021 (Sun et al., 2022). As a group of chronic metabolic disorders resulting from dysfunction or progressive loss of the insulin-producing β cells residing in the pancreatic islets, diabetes can lead to various severe complications, including kidney failure, coronary artery disease, stroke, and even premature death. Type 1 diabetes is caused by a dysregulated autoimmune reaction towards pancreatic β cells, while type 2 diabetic patients suffer an insulin action deficiency caused by pancreatic β cell dysfunction and peripheral insulin resistance (Katsarou et al., 2017; Chatterjee et al., 2017). In addition, there are a variety of rare monogenic diabetes, including neonatal diabetes that manifest at birth and maturity-onset diabetes of the young (MODY), which result from mutations in a single gene critical for pancreatic β cell development and/or function (Flannick et al., 2016). Currently, the approaches to alleviate type 1 diabetes mainly rely on exogenous insulin injection. Keeping a healthy diet and weight, taking oral antidiabetics, or even injecting insulin is available for type 2 diabetes treatments. Nonetheless, these treatments are difficult to mimic the in vivo accurate glucose control and may lead to hypoglycemia or hyperglycemia, further increasing the risk of other complications. Transplantation of cadaveric pancreas or islets has been used to treat diabetes; however, it is limited by the insufficient organ donor and risk of immune rejection (Ryan et al., 2005; Farney et al., 2016). Therefore, the alternative source of stem cell-derived pancreatic β cells holds promise for solving these problems.

Stem cells are a kind of unspecialized or partially specialized cells with self-renewal and multilineage differentiation potential. Compared to those tissue stem cells existing in adult tissues or organs, embryonic stem cells (ESCs) derived from the inner cell mass of blastocysts are pluripotent which can form three germ layers (ectoderm, mesoderm, and endoderm) (Thomson et al., 1998). In addition to ESCs, human-induced pluripotent stem cells (iPSCs), reprogrammed from human somatic cells through the ectopic expression of four transcription factors (OCT3/4, KLF4, SOX2, and c-MYC), are pluripotent as well and share the similar signatures in morphology, transcriptome and epigenome to ESCs (Takahashi et al., 2007). Both pluripotent stem cells (PSCs) can theoretically proliferate indefinitely in vitro while maintaining the capacity to differentiate into all cell types of the three germ layers. Notably, iPSCs can be individualized for patients to potentially protect from immune attacks. Alternatively, other PSC types with advanced developmental potential show promise. For instance, naïve PSCs or even totipotent-like stem cells have recently been established (Guo et al., 2016; Geng et al., 2019; Mazid et al., 2022). The human expanded potential stem cells or extended pluripotent stem cells (EPSCs) exhibit both embryonic and extraembryonic bidirectional developmental potential (Yang et al., 2017; Gao et al., 2019; Zheng et al., 2021), possibly providing an alternative cell source. Thus, human PSCs could be feasible to manufacture limitless numbers of the human cells with given types in vitro for transplantation therapies, including pancreatic β cells and other diabetes-relevant cells (Kim et al., 2020).

**Fig. 1:**
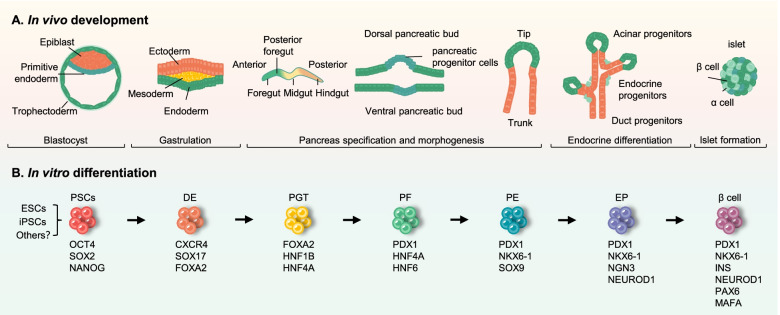
Stepwise differentiation of pancreatic β cells from human pluripotent stem cells. **A** Pancreatic islets are derived from the definitive endoderm, which is specified during gastrulation and then forms the primitive gut tube. During the development from the foregut to the pancreatic endoderm, pancreatic buds consisting of pancreatic progenitor cells emerge from the dorsal and ventral sides of the posterior foregut, following that the pancreatic epithelium expands and differentiates into endocrine progenitor cells, which finally give rise to β cells. **B** Through mimicking in vivo pancreatic development, human pluripotent stem cells (PSCs) are differentiated stepwise into pancreatic lineage and eventually to generate β cells. DE: Definitive Endoderm; PGT: Primitive Gut Tube; PF: Posterior Foregut; PE: Pancreatic Endoderm; EP: Endocrine Precursor

Understanding the morphogenesis and expression profiles of key lineage-specific genes during pancreatic development provides a basis for the in vitro pancreatic β cells differentiation from stem cells (Schwitzgebel et al., 2000; Wilson et al., 2003; Jennings et al., 2015). Specifically, pancreatic islets are derived from the definitive endoderm specified during gastrulation. The endoderm then folds to form the primitive gut tube, followed by independent budding of the dorsal and ventral buds at the posterior region of foregut, and then two buds grow and eventually fuse to form the pancreatic endoderm after gut rotation. Pancreatic epithelium, consisting of multipotent progenitor cells, morphologically transforms and forms exocrine and endocrine components. The endocrine cells are further specified into insulin-producing pancreatic β cells, glucagon-producing α cells, somatostatin-producing δ cells, pancreatic polypeptide-producing PP cells, and ghrelin-producing ε cells (Benitez et al., 2012; Walker et al., 2021). Mimicking in vivo pancreatic development by sequential supplementation of small molecules and growth factors, stepwise directed differentiation enables the progression of human PSCs from the pluripotent stage toward the pancreatic lineage and finally to insulin-producing pancreatic β cells (Al-Khawaga et al., 2018). Briefly, cultured human PSCs need to be induced into definitive endoderm first; subsequently, the endodermal cells would be specialized into pancreatic progenitors; through the stage of pancreatic endocrine progenitors, these cells are directed to a pancreatic β cell fate lastly (Fig. 1).

## Induction of definitive endoderm

Definitive endoderm forms during gastrulation. At about 14 days post conception (dpc) of human embryonic development, the epiblast cells undergo the epithelial-mesenchymal transition to specify into primitive streak, followed with endoderm and mesoderm formation. The definitive endoderm develops from the specific anterior region of primitive streak, which is mostly regulated by the transforming growth factor-β (TGF-β) superfamily (including Nodal/Activin A signaling and bone morphogenetic protein (BMP) signaling) and Wingless and Int-1 (WNT) signaling. In vitro, human PSCs can correspondingly differentiate into mesendoderm (equivalent to the primitive streak) and subsequent mesoderm or endoderm lineage.

D'Amour and colleagues demonstrated the differentiation of human ESCs into definitive endoderm by using Activin A for mimicking the function of Nodal (D'Amour et al., 2005). The efficiency of mesendoderm specification and the synchrony of definitive endoderm formation can be improved by the combined activation of WNT signaling by WNT3A (D'Amour et al., 2006). Furthermore, it was shown that the combination of Activin A or GDF8 (a TGF-β family member) with CHIR99021, an inhibitor of glycogen synthase kinase 3-β (GSK3-β) as well as an activator of the canonical WNT/β-catenin pathway could induce definitive endoderm more effectively than the combination with WNT3A (Rezania et al., 2014; Bruin et al., 2014). Specifically, high Nodal/Activin A plus WNT, BMP, or fibroblast growth factor (FGF) signaling induces the differentiation of human PSCs into mesendoderm; subsequently, since WNT signaling pathway promotes primitive streak to mesoderm lineage and represses endoderm differentiation, the mesendoderm cells are differentiated into definitive endoderm by high Nodal/Activin A and withdrawal of WNT (Jiang et al., 2013). Additionally, active phosphoinositide 3 kinase (PI3K) signaling inhibits definitive endoderm specification, and PI3K inhibitors such as LY294002 (McLean et al., 2007) and Wortmannin (Zhang et al., 2009) could be used to improve the yield of definitive endoderm. Recently, Jun N-terminal kinase (JNK)-JUN signaling was found to be a barrier to human PSC-derived definitive endoderm differentiation, which would inhibit the generation of pancreatic progenitor cells in the following process from human PSCs to definitive endoderm differentiation (Li et al., 2019).

Current protocols can yield more than 90% of cells expressing definitive endoderm markers FOXA2, SOX17, and CXCR4, but this population is highly heterogeneous with different differentiation potency to various endodermal lineages such as liver, pancreas, and lung. Very interestingly, it was found that CD177-positive definitive endoderm subpopulation was permissive to the pancreatic fate, while CD275-positive definitive endoderm subpopulation was specified toward the hepatic fate (Mahaddalkar et al., 2020).

## Specialization into pancreatic progenitor cells

After the endoderm germ layer arises, the definitive endoderm is subsequently surrounded by mesoderm and undergoes a series of morphological changes to form the primitive gut tube, which can be achieved by using FGF10 or keratinocyte growth factor (KGF) (Kroon et al., 2008; Pagliuca et al., 2014). The primitive gut tube is segmented along the anterior–posterior axis into the anterior endoderm (foregut) and posterior endoderm (midgut/hindgut), giving rise to the various endoderm-derived organs. The midgut and hindgut regions give rise to the small intestine and colon, while the foregut develops into the lung, esophagus, thyroid, stomach, liver, and pancreas. The pancreas first emerges as buds from the dorsal and ventral sides of the posterior foregut, wherein the dorsal bud appears first at about 29 dpc while the ventral bud appears at about 30 dpc. The two pancreatic buds, consisting of pancreatic progenitor cells expressing PDX1, subsequently elongate alongside the presumptive duodenum and stomach and eventually fuse together at 37 dpc through gut rotation, wherein the pancreatic progenitor cells form a multilayered epithelium with a centrally located lumen. The pancreatic epithelium continues to expand and branch into an epithelial tree-like tubular network, progressively segregate into tip (expressing PTF1A, CPA1, etc.) or trunk domains (expressing NKX6-1, SOX9, etc.) and differentiate into acinar or bipotent endocrine/duct progenitor cells respectively. PDX1 promotes the expansion and differentiation of pancreatic progenitors in concert with NKX6-1, which is expressed in pancreatic buds from about 30 dpc to 40 dpc and is restricted to β cells later (Bastidas-Ponce et al., 2017; Larsen and Grapin-Botton, 2017).

The commitment of the foregut into pancreatic lineage is mediated by signaling, including sonic hedgehog (SHH), retinoic acid (RA), FGF, and BMP. SHH blocks the differentiation of dorsal pancreatic buds, and inhibition of SHH signaling promotes pancreatic lineage development in mice and humans (Hebrok et al., 1998; Kim and Melton, 1998). RA plays a crucial role in the induction of PDX1 expression (Martin et al., 2005; Molotkov et al., 2005; Jiang et al., 2007). However, retinoid receptors are upregulated in the pancreatic exocrine (Kadison et al., 2001). Therefore, the dose of RA is progressively lowered in most in vitro differentiation protocols: high concentration induces PDX1 expression while low concentration maintains PDX1 and induces/maintains NKX6-1 expression (Rezania et al., 2014; Pagliuca et al., 2014). Moreover, FGF signaling from the cardiac mesoderm leads to the induction of the hepatic lineage and segregation of pancreas and hepatobiliary progenitors (Deutsch et al., 2001). FGF signaling from pancreatic mesenchyme contributes to the growth and branching of the pancreatic epithelium (Bhushan et al., 2001; Gnatenko et al., 2017). BMP signaling has been demonstrated to segregate the hepatic and pancreatic lineages and induce liver formation (Rossi et al., 2001; Nostro et al., 2011; Lee et al., 2021). Besides, protein kinase C (PKC) agonists enhance PDX1 expression and promote the generation of NKX6-1-positive cells while minimizing the formation of intestinal and hepatic lineages (Chen et al., 2009; Rezania et al., 2012; Pagliuca et al., 2014). Epidermal growth factor (EGF) promotes the expansion of pancreatic progenitors by improving the number of PDX1-positive cells (Zhang et al., 2009). In addition, modulation of WNT signaling promotes pancreatic lineage differentiation (Sharon et al., 2019; Tan et al., 2019; Mahaddalkar et al., 2020).

Notably, PDX1-positive/NKX6-1-negative cells are found to differentiate into non-functional polyhormonal cells (Aigha and Abdelalim, 2020), while pancreatic progenitor cells co-expressing PDX1 and NKX6-1 derived from human PSCs could mature into functional β cells in vivo when transplanted into mice (Kroon et al., 2008). Consequently, sufficient NKX6-1-positive cells are critical for the eventual generation of insulin-producing pancreatic β cells. Nostro and colleagues demonstrated that the combination of EGF and nicotinamide signaling efficiently induced the generation of NKX6-1-positive progenitors from multiple human PSC lines (Nostro et al., 2015). Moreover, the percentage of the NKX6-1-positive population was regulated by the duration of RA/FGF10 induction and inhibition of BMP and SHH signaling pathways (Nostro et al., 2015). Furthermore, dissociation of densely formed definitive endoderm cells and re-plating them at low density resulted in a high yield of pancreatic progenitors co-expressing PDX1 and NKX6-1 (Memon et al., 2018). Very recently, Liu and colleagues identified ten chemicals (LDN, T3, SANT1, Repsox, RA, ZnSO_4_, TPB, EGF, Nicotinamide, and GABA) that retained pancreatic progenitor cells in 3D clusters and boosted their potency for NKX6-1 and insulin co-expressing β cells. With combinations of signaling pathway regulators for later step, eventually resulting in the generation of β cells with high efficiency (Liu et al., 2021). Intriguingly, a novel population of pancreatic progenitors expressing NKX6-1 but not PDX1 has been identified during in vitro pancreatic differentiation from human PSCs (Memon et al., 2018; Aigha et al., 2018), which can further differentiate into glucose-responsive INS-positive β cells in vitro (Memon et al., 2021). These PDX1-negative/NKX6-1-positive cells are non-epithelial with high expression of NESTIN but lacking the pancreatic epithelial marker CDH1, and similar to progenitor cells resident in the pancreatic mesenchyme, indicating that there is an alternative β cell determination route distinct from that of the PDX1 and NKX6-1 co-expressing progenitors during development (Memon et al., 2021).

## Differentiation of endocrine progenitors

The bipotent trunk epithelium expressing NKX6-1 and SOX9 differentiates to endocrine progenitor cells under activation of NGN3, a pro-endocrine transcription factor (Gu et al., 2002). During mouse embryonic development, there is a biphasic transient wave of NGN3 expression: the first wave of NGN3-positive cells appears at around embryonic day (E) 9.0, and the second wave peaks at E15.5 (Villasenor et al., 2008). However, there is only a single wave of NGN3 expression peaking at 10–14 weeks post conception (wpc) in humans (Salisbury et al., 2014). NGN3 is expressed at low levels in cell-cycle-active bipotent progenitors within the trunk epithelium, and the transient increase in NGN3 levels triggers endocrine commitment.

Notch signaling plays a critical role in the specification of epithelial cells towards bipotent ductal cells or endocrine progenitors. Suppression of Notch signaling promotes the expression of NGN3 and the specification of endocrine progenitors. Notch signaling inhibitors, including gamma-secretase inhibitor DAPT, XXI, and YO-01027, promote the expression of pancreatic endocrine lineage markers (D'Amour et al., 2006; Rezania et al., 2014; Pagliuca et al., 2014). Inhibition of BMP and TGF-β is required for efficient endocrine development as well (Nostro et al., 2011). The addition of the TGF-β receptor inhibitor Alk5iII and thyroid hormone can transiently upregulate the expression of NGN3.

Temporal regulation of NGN3 induction is critical for the specification of endocrine cells (Zhu et al., 2016). Precocious induction of NGN3 results in the production of polyhormonal cells (Russ et al., 2015). Canonical WNT signaling was found to inhibit pancreatic differentiation, whereas noncanonical WNT signaling promoted pancreatic fate. Meanwhile, noncanonical WNT signaling promoted cell cycle exit, while the prolongation of the cell cycle was crucial to NGN3 induction (Mahaddalkar et al., 2020). Additionally, YAP is involved in the activation of the key pancreatic program and regulates the expansion of pancreatic progenitor cells (Cebola et al., 2015). YAP1 deletion directs pancreatic progenitors to the endocrine lineage both in vivo and in vitro, resulting in increased NGN3 and insulin expression levels (Mamidi et al., 2018). Inhibition of YAP reduces the proliferation of pancreatic progenitor cells, enhancing the differentiation of endocrine progenitor cells and the formation of β cells (Rosado-Olivieri et al., 2019).

## Maturation of pancreatic β cells

Endocrine progenitors expressing a high level of NGN3 are subsequently committed to distinct endocrine cell types under the expression of different lineage-specific transcription factors. In humans, the first fetal β cells emerge at about 8 wpc, followed by the formation of glucagon-producing α cells at 9 wpc, whereas in mice α cells appear earlier than β cells (Jennings et al., 2015). Endocrine progenitor cells, highly expressing NEUROD1, ISL1, NKX2-2, and PAX6 during 8 and 12 weeks, are specified as β cells together with PDX1 and NKX6-1 expression (Lyttle et al., 2008). These β cells, which have high proliferation and increased basal rate of insulin secretion, are immature until postnatal periods (Henquin and Nenquin, 2018). After birth, the expression of insulin transcription-and secretion-related genes, including *NEUROD1*, *PAX6*, *MAFA*, *PCSK1/3*, *ABCC8*, *SLC30A8*, *GCK*, and *GLUT1* (Lemaire et al., 2016; Campbell and Newgard, 2021), enables the β cells to respond to high glucose levels with an appropriate insulin release, the hallmark of mature β cells. NEUROD1 is required for β cell maturation and maintenance of glucose-responsive capacity (Gu et al., 2010). PAX6 is an activator of several β-cell genes involved in insulin synthesis, glucose sensing, and insulin secretion (Swisa et al., 2017; So et al., 2021). MAFA activates insulin gene transcription and regulates the expression of genes involved in insulin biosynthesis and secretion, which is considered as the key to establishing mature functional β cells (Zhang et al., 2005; Wang et al., 2007). Furthermore, PCSK1/3 is involved in the process of proinsulin to insulin; *ABCC8* encodes potassium channel-associated receptors; SLC30A8, GCK, and GLUT1 are associated with glucose transport (Krentz and Gloyn, 2020). Likewise, the disallowed genes such as *SLC16A1*(encoding monocarboxylic acid transporter 1) and *LDHA* (encoding lactate dehydrogenase A), which are highly expressed in neonatal β cells need to be repressed for β cell maturation (Pullen et al., 2010; Lemaire et al., 2016). In addition, the β cells further sense the environmental signals and adjust the insulin secretory response for glucose control (Wortham and Sander, 2021).

Most of the pancreatic differentiation protocols from endocrine progenitor cells to β cells primarily involve modulation of TGF-β signaling, thyroid hormone, and gamma-secretase (Rezania et al., 2014; Pagliuca et al., 2014). TGF-β superfamily members play important roles in regulating β cell development and function (Lee et al., 2021). Thyroid hormones regulate insulin secretion, possibly by controlling glucose oxidation and calcium uptake rates (Cortizo et al., 1985). T3, a kind of thyroid hormone, can enhance insulin signaling and increase insulin synthesis (Goulart-Silva et al., 2012; Verga Falzacappa et al., 2010). Alk5iII maintains NKX6-1 expression in endocrine cells, and T3 increases insulin expression in the NKX6-1-expressing endocrine cells, both together improve the function of PSC-derived β cells. The addition of gamma-secretase inhibitor XX (GSiXX) in concert with T3 increases NKX6-1 and insulin co-expressing β cell production. Rezania and colleagues further screened R428, an inhibitor of tyrosine kinase receptor AXL, which could effectively upregulate the expression of MAFA together with the introduction of N-acetyl cysteine (Rezania et al., 2014). The resulting pancreatic β cells can secrete insulin in response to glucose stimulation and reverse hyperglycemia in diabetic mice (Rezania et al., 2014; Pagliuca et al., 2014).

Nonetheless, the current human PSC-derived pancreatic β cells generated in vitro are generally evaluated by the capability to secrete insulin and express a set of β cell identity genes, which are not sufficient for the function of glucose-stimulated insulin secretion (GSIS). Those cells are transcriptionally and functionally immature compared to primary pancreatic β cells, and their function and maturity are mainly dependent on the in vivo environment after transplantation (Augsornworawat et al., 2020). Treatment with the ROCK inhibitor H1152 promotes the maturation of β cells, with increased *MAFA* and *UCN3* expression (Ghazizadeh et al., 2017). The addition of noncanonical WNT signaling WNT4 promotes metabolic maturation of β cells and robust GSIS (Yoshihara et al., 2020). Significantly, removing the inhibitor Alk5iII that is used in many protocols at final stage (Rezania et al., 2014; Pagliuca et al., 2014), controlling cellular cluster size, and using a serum-free media were found to be able to generate β cells that can undergo biphasic dynamic GSIS (Velazco-Cruz et al., 2019; Nair et al., 2019).

## Concerns and recent advances in stepwise pancreatic β cell differentiation

Despite the fact that great accomplishments have been made in the stepwise differentiation of human PSCs into pancreatic β cells, there are still several concerns, such as low efficient differentiation, limited functional maturity of β cells, and poor glucose-stimulated insulin secretion.

### Differentiation efficiency

**Fig. 2:**
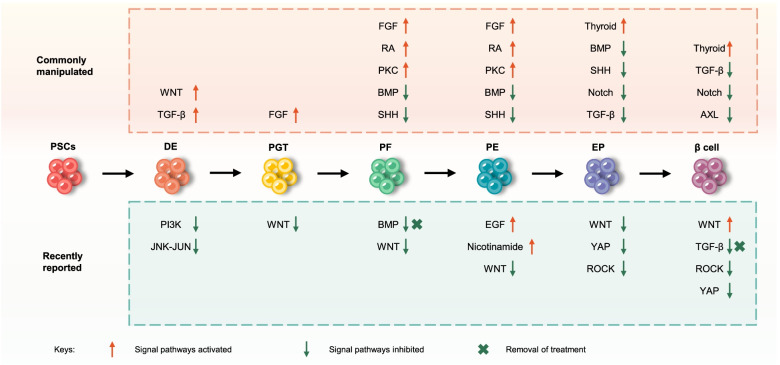
Improvements to efficient differentiation of human PSC-derived pancreatic β cells. The current improvements of efficient differentiation mainly focus on exploring signal pathways. Key signal pathways and related molecules are shown in the graph. The top half of the figure depicts the commonly manipulated signaling pathways; the bottom half shows the recently reported signaling pathways or molecules improving differentiation efficiency

In addition to exploring signal pathways or small molecules summarized above, current improvements in efficient differentiation also have paid attention to the enrichment of certain cell populations and optimization of culture systems (Fig. 2). Purification and enrichment by cell-surface marker have been used to improve the efficiency and purity of pancreatic differentiation. Enrichment of anterior definitive endoderm cells by the surface marker CD177/NB1 glycoprotein increases the efficiency of pancreatic differentiation and functional maturation of β cells (Mahaddalkar et al., 2020). CD24, CD142, and Glycoprotein 2 were found as a cell-surface marker for pancreatic progenitor cells (Jiang et al., 2011; Kelly et al., 2011; Ameri et al., 2017; Cogger et al., 2017). CD49a (also known as ITGA1) was identified as a marker for PSC-derived β cells (Veres et al., 2019). CD9 was found as a negative cell-surface marker for β cells (Li et al., 2020). Furthermore, a recent study identified three monoclonal antibodies to mark islet endocrine cell populations, which were used for magnetic sorting to enrich insulin-expressing cells with a high fraction of recovery (Parent et al., 2022).

Alternatively, manipulating the architecture and substrate stiffness of culture platforms to mimic biophysical features of the developmental microenvironment also contributes to pancreatic differentiation (Tran et al., 2020; Jiang et al., 2022). Air–liquid interface culture (Rezania et al., 2014) or suspension culture (Pagliuca et al., 2014) allows closer mimicry of in vivo development. Very recently, Goncalves and colleagues developed a three-dimensions culture system that allowed the self-organization and long-term expansion of pancreatic progenitors, and the resulting pancreatic progenitors were transcriptionally closer to the fetal pancreas (Goncalves et al., 2021). Liu and colleagues also highlighted that poising the pancreatic progenitors with an extended three-dimensions culture during the differentiation process in case of the premature loss of the progenitor state enhanced their potential to differentiate into β cells (Liu et al., 2021). High stiffness of tissue culture polystyrene induced actin polymerization that prevented premature NGN3 expression and promoted NKX6-1 expression but also inhibited further differentiation into the endocrine lineage. Treatment of latrunculin A to depolymerize the cytoskeleton during endocrine induction could efficiently generate PSC-derived β cells in two-dimensional culture. Meanwhile, the resulting β cells reversed diabetes within two weeks after being transplanted into streptozotocin-treated mice, whereas suspension culture took about three weeks (Hogrebe et al., 2020).

### Functional maturation

**Fig. 3:**
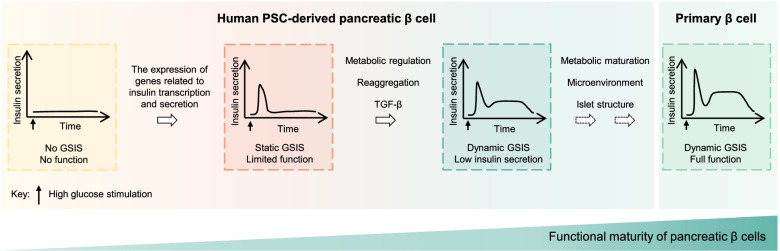
Efforts on the functional maturation of human PSC-derived pancreatic β cells. The pancreatic β cells derived from human PSCs showed limited functional maturity. The expression of genes related to insulin transcription and secretion enables immature β cells to respond to high glucose levels with an appropriate insulin release, showing static GSIS with limited function. Dynamic GSIS with first- and second-phase insulin secretion has been achieved by regulating TGF-β signal pathway, reaggregation and/or metabolic regulation, but the amount of insulin secreted in the second stage is still low. Metabolic maturation of β cells, together with the microenvironment and islet structure considerations contribute to the functional maturity of human PSC-derived pancreatic β cells. GSIS: glucose-stimulated insulin secretion

Limited functional maturity of differentiated β cells, manifesting as the production of polyhormonal cells and poor GSIS, is a key unsolved problem in pancreatic differentiation. During pancreas organogenesis, endocrine cells detach from the pancreatic epithelium lumen and cluster into islet-like structures to acquire functional maturity (Bastidas-Ponce et al., 2017). Mimicking endogenous endocrine cells clustering by isolating and reaggregating PSC-derived immature β cells induces β cell maturation, which displays many physiological properties of adult human islets, including dynamic insulin secretion, calcium signaling, highly sensitive K^+^-ATP channels, and mitochondrial energization (Nair et al., 2019). Mechanistically, immature β-like cell reaggregation induces metabolic mitochondrial remodeling enabling β-cell functional maturation. Indeed, a recent report also reveals that mitochondrial remodeling is required for proper definitive endoderm differentiation from human PSCs, while ATP greatly facilitates the differentiation process (Lv et al., 2022). Three-dimensional architecture and WNT/PCP pathway activation could induce β cell maturation and increase GSIS (Bader et al., 2016). WNT4 promotes metabolic maturation of β cells and robust GSIS, which dose-dependently increases the expression of *ESRRG* (encoding ERRγ) and components of the mitochondrial respiratory chain *NDUFA7* and *COX7A2* (Yoshihara et al., 2020). Consistently, ERRγ overexpression directs metabolic maturation in human iPSC-derived β cells (Yoshihara et al., 2016), indicating that WNT4 is likely to drive the metabolic maturation of β cells through the induction of an ERRγ gene network. Taken together, the metabolic switch from glycolysis to mitochondrial respiration is essential for β cell maturation and normal physiological function, which can be improved by the reaggregation of endocrine cells and modulation of WNT signaling. In addition, regulation of nutrient-sensing by mTORC1 enhanced glucose-responsive insulin secretion (Helman et al., 2020). Functional maturation of β cells can be improved through controlling energy metabolism and GSIS function by circadian modulation (Alvarez-Dominguez et al., 2020) (Fig. 3).

## Clinical applications of PSC-derived pancreatic lineages

Human PSC-derived pancreatic progenitors could mature into functional β cells when transplanted into mice and eventually reverse diabetes (Kroon et al., 2008; Szot et al., 2015). Besides the mouse model, a recent report showed that the human PSC-derived β cells could rescue hyperglycemia in non-human primates (Du et al., 2022). The pancreatic lineages (pancreatic progenitors or β cells) derived from human PSCs present the potential in cell replacement therapies for diabetes mellitus. In fact, progress has been made in phase 1/2 clinical trials for PSC-based islet replacement therapy, and current efforts are mainly focused on type 1 diabetes.

Viacyte (previously known as NovoCell) launched a clinical trial in 2014 to evaluate the therapeutic efficacy of human ESC-derived pancreatic progenitors that were considered to further differentiate into β cells and other islet cells through in vivo maturation after transplantation in patients with type 1 diabetes. The pancreatic progenitor cells were placed in an immunoprotective macroencapsulation device that allowed free transport of oxygen, nutrients, and proteins and implanted subcutaneously (ClinicalTrials.gov NCT02239354). However, there was no insulin secretion despite the insulin-positive cells being detected in several explanted grafts 24 weeks post-implantation, and the cell death due to device fibrosis and lack of efficient vascularization could not be ignored (Henry et al., 2018). A subsequent trial used a non-immunoprotective macroencapsulation device that allowed blood vessels to enter the device and directly contact the graft cells to improve the cell survival (ClinicalTrials.gov NCT03163511). The results showed that the patients developed a mixed meal-stimulated C-peptide secretion after transplantation. Nonetheless, the insulin production did not reach therapeutic levels and there was no clinically relevant effect. In addition, the glucagon-expressing α cells were predominant in the explants, while β cells were a small minority (Ramzy et al., 2021; Shapiro et al., 2021). The human ESC-derived pancreatic progenitors are expandable and cryopreserved in vitro (Trott et al., 2017; Nakamura et al., 2022), and express relatively low levels of human leukocyte antigens (HLAs) (van der Torren et al., 2017). However, it takes time to differentiate into β cells after partially differentiated pancreatic progenitor cells transplantation and the in vivo environment has potential influence on the differentiation preference of pancreatic progenitor cells (Bruin et al., 2016; Saber et al., 2018), whereas transplantation of β cells could be a one-step approach and the ultimate choice for diabetes therapy. Following the advances in the differentiation of human PSCs into pancreatic β cells, a clinical trial using human PSC-derived β cells VX-880 infusion is ongoing, which is the first to use differentiated β cells for the diabetes therapy (ClinicalTrials.gov NCT04786262).

The current phase 1/2 clinical trials of PSC-based islet replacement therapy require long-term combined immunosuppressive therapy. One reason is the immune response caused by allograft, and another major concern is that patients with type 1 diabetes have severe autoimmunity, which would destroy the transplanted β cells as well. It is obvious that long-term immunosuppression could increase the risk of infection and cancer development (Fishman, 2017). However, the way to protect grafts from immune rejection in immune-protective macroencapsulation devices usually limits the efficient vascularization of the grafts and causes the death of β cells (ClinicalTrials.gov NCT02239354). The ideal islet encapsulation device needs to keep sufficient grafts functional to regulate blood glucose, be biocompatible, insulate grafts from the immune system, and make nutrition and oxygen available for maintaining cell viability (Korsgren, 2017; Jiang et al., 2022). Optimization of cell encapsulation strategy shows promise for circumventing immunosuppressive therapy (Desai and Shea, 2017; Goswami et al., 2021). For example, the human PSC-derived β cells encapsulated with triazole-thiomorpholine dioxide alginate showed long-term glycemic control and mitigated immune response after transplantation into immunocompetent mice (Vegas et al., 2016). Microencapsulation of human PSC-derived β cells with alginate incorporating high-dose cytokine CXCL12 prolonged glycemic correction in immunocompetent diabetic mice without systemic immunosuppression (Alagpulinsa et al., 2019). Some nanofiber-based encapsulation devices also present the potential for immune protection (An et al., 2018; Wang et al., 2021). Additionally, modulating the immune response to generate immune-evasive human PSC-derived pancreatic lineage grafts presents another contribution to reduce alloimmunity and autoimmunity. Overexpression of PD-L1 or treatment with interferon γ reduced immunogenicity of human PSC-derived β cells and protected them from graft rejection after transplantation into mice (Yoshihara et al., 2020). Depletion of the HLAs, the main drivers of allogeneic rejection, and retention of HLA-A2, and less polymorphic HLA alleles, protected human PSC-derived β cells from T-cell-mediated rejection in a humanized mouse model (Parent et al., 2021). Undoubtedly, the immune-invasive grafts would carry risks such as neoplastic growths, pathogenic infections, and other complications. Cai and colleagues demonstrated that the deletion of *RNLS*, a risk gene for type 1 diabetes, endowed β cells with the ability to resist autoimmune killing in both mice and humans (Cai et al., 2020). Besides, generating patient-specific iPSC could significantly alleviate immune rejection, but this personalized treatment is expensive. The establishment of universal iPSC lines with the HLA types matching the majority of potential recipients provides a feasible approach (Yamanaka, 2020).

## Conclusions

To date, significant advances have been made in stepwise pancreatic differentiation. Indeed, pancreatic β cell differentiation efficiency of human PSCs has achieved up to 40%-70% without sorting or enrichment (Rezania et al., 2014; Pagliuca et al., 2014; Velazco-Cruz et al., 2019; Hogrebe et al., 2020; Liu et al., 2021) (Table 1). However, the current protocols are not reproducible enough, and different human PSC lines exhibit variable differentiation efficiency (Merkle et al., 2022). Indeed, efficient differentiation of different PSC lines usually requires precise controlling of the seeding density and signal activities. Compared with ESCs, there are fewer ethical concerns and immunogenicity for iPSC-based islet replacement, but iPSCs face the risk of mutation from reprogramming (Miura et al., 2009; Yamanaka, 2020), and the differentiation efficiency is generally more variable (Kyttala et al., 2016; Carcamo-Orive et al., 2017). Furthermore, given that the pluripotency heterogeneity could result in variable differentiation efficiency (Li and Izpisua Belmonte, 2018), other PSC types with advanced differentiation propensity, such as the naïve PSCs, totipotent-like stem cells, and EPSCs, likely contribute to overcoming this obstacle. Supporting this notion, human EPSCs have been manifested to differentiate into functional hepatocytes with higher efficiency; furthermore, the gene expression profile of EPSC-derived hepatocytes is more similar to human primary hepatocytes than the iPSC derivates (Wang et al., 2020). This provides a feasible direction to improve the efficiency, robustness, and maturity of pancreatic β cell differentiation.

**Table 1:** Overview of recent protocols for stepwise differentiation of pancreatic β cells from human PSCs

Work	Differentiation protocol		Cell line used	Culture format	Reaggregation/ Enrichment	Differentiation efficiency		
						**% Pancreatic progenitors**	**% Endocrine progenitors**	**% β cells**
(Rezania et al., 2014)	Stage 1: DE	GDF8 + MCX-928/CHIR	H1	Planar culture/air–liquid interface	No/ No	~ 60% PDX1^+^/NKX6-1^+^	~ 42% NKX6.1^+^/CHGA^+^	~ 50% insulin^+^/NKX6-1^+^
	Stage 2: PGT	FGF7						
	Stage 3: PP1	FGF7 + RA + TPB + LDN + SANT1						
	Stage 4: PP2	FGF7 + RA + TPB + LDN + SANT1						
	Stage 5: EP	RA + SANT1 + ALK5iII + T3 + LDN						
	Stage 6: IB	ALK5iII + T3 + LDN + GSiXX						
	Stage 7: β cell	ALK5iII + T3 + N-Cys + R428						
(Pagliuca et al., 2014)	Stage 1: DE	Activin A + CHIR	HUES8	Suspension culture	No/ No	> 55% PDX1^+^/NKX6-1^+^	n.d	33% ± 2% C-peptide^+^/ NKX6-1^+^
	Stage 2: PGT	KGF						
	Stage 3: PP1	KGF + RA + PdBU + LDN + SANT1						
	Stage 4: PP2	KGF + RA + SANT1						
	Stage 5: EP	RA + SANT1 + ALK5iII + T3 + XXI + Betacellulin						
	Stage 6: β cell	ALK5iII + T3						
(Russ et al., 2015)	Stage 1: DE	Activin A + WNT3A	MEL1- INS^GFP/W^	Low-adherence plates	No/ No	~ 90% PDX1^+^/NKX6-1^+^	n.d	17% ± 6% C-peptide^+^/ NKX6-1^+^
	Stage 2: PGT	KGF + TGF-βiIV						
	Stage 3: PP1	RA						
	Stage 4: PP2	EGF + KGF						
	Stage 5: EP	TPB + ALK5iII + Noggin + KGF						
	Stage 6: β cell	No factors						
(Millman et al., 2016)	Stage 1: DE	Activin A + CHIR	ND (non-diabetic) iPSC; T1D (Type 1 diabetic) iPSC	Suspension culture	No/ No	(ND) 52% -79% PDX1^+^/NKX6-1^+^ (T1D) 59% -88% PDX1^+^/NKX6-1^+^	n.d	(ND)27% ± 2% C-peptide^+^/ NKX6-1^+^ (T1D)24% ± 2% C-peptide^+^/ NKX6-1^+^
	Stage 2: PGT	KGF						
	Stage 3: PP1	KGF + RA + PdBU + LDN + SANT1 + Y27						
	Stage 4: PP2	KGF + RA + SANT1 + Activin A + Y27						
	Stage 5: EP	RA + SANT1 + ALK5iII + T3 + XXI + Betacellulin						
	Stage 6: β cell	ALK5iII + T3						
(Ghazizadeh et al., 2017)	Stage 1: DE	Activin A + CHIR	H1; HUES8; HES3- INS^GFP/W^	Planar culture/air–liquid interface	No/ No	n.d	n.d	34% C-peptide^+^
	Stage 2: PGT	FGF7						
	Stage 3: PP1	FGF7 + RA + TPB + LDN + SANT1						
	Stage 4: PP2	FGF7 + RA + TPB + LDN + SANT1						
	Stage 5: EP	RA + SANT1 + ALK5iII + T3 + LDN						
	Stage 6: IB	ALK5iII + T3 + LDN + GSiXX						
	Stage 7: β cell	ALK5iII + T3 + LDN + H1152						
(Velazco-Cruz et al., 2019)	Stage 1: DE	Activin A + CHIR	HUES8	Suspension culture	Stage 6/ No	n.d	96% ± 1% CHGA^+^	52% C-peptide^+^/ NKX6-1^+^
	Stage 2: PGT	KGF						
	Stage 3: PP1	KGF + RA + PdBU + LDN + SANT1 + Y27						
	Stage 4: PP2	KGF + RA + SANT1 + Activin A + Y27						
	Stage 5: EP	RA + SANT1 + ALK5iII + T3 + XXI + Betacellulin						
	Stage 6: β cell	ESFM						
(Veres et al., 2019)	Stage 1: DE	Activin A + CHIR	HUES8	Suspension culture	Stage 6/ CD49a^+^ at Stage 6	n.d	~ 95% CHGA^+^	80% C-peptide^+^/ NKX6-1^+^
	Stage 2: PGT	KGF						
	Stage 3: PP1	KGF + RA + PdBU + LDN + SANT1 + Y27						
	Stage 4: PP2	KGF + RA + SANT1 + Activin A + Y27						
	Stage 5: EP	RA + SANT1 + ALK5iII + T3 + XXI + Betacellulin						
	Stage 6: β cell	No factors						
(Rosado-Olivieri et al., 2019)	Stage 1: DE	Activin A + CHIR	HUES8	Suspension culture	No/ No	~ 43.6% PDX1^+^/NKX6-1^+^	12.1 ± 2% NGN3^+^	38.6 ± 3.9% C-peptide^+^/ NKX6-1^+^
	Stage 2: PGT	KGF						
	Stage 3: PP1	KGF + RA + PdBU + LDN + SANT1						
	Stage 4: PP2	KGF + RA + SANT1 + Activin A + Y27						
	Stage 5: EP	RA + SANT1 + ALK5iII + T3 + XXI + Betacellulin + verteporfin						
	Stage 6: β cell	verteporfin						
(Nair et al., 2019)	Stage 1: DE	Activin A + WNT3A	MEL1- INS^GFP/W^	Suspension culture	Stage 6/ INS: GFP; at Stage 6	> 70% PDX1^+^/NKX6-1^+^	99% CHGA^+^	85% C-peptide^+^/ NKX6-1^+^
	Stage 2: PGT	KGF + TGF-βiIV						
	Stage 3: PP1	TTNPB						
	Stage 4: PP2	TTNPB + EGF + KGF						
	Stage 5: EP	ALK5iII + T3 + LDN + XXI						
	Stage 6: β cell	ALK5iII + T3						
(Mahaddalkar et al., 2020)	Stage 1: DE	Activin A + WNT3A	H1; H9; HUES8; MEL1-NKX6.1^GFP^	Suspension culture	Stage 3/ CD177^+^ at Stage 1	~ 60% PDX1^+^/NKX6-1^+^	n.d	~ 62% insulin^+^/NKX6-1^+^
	Stage 2: PGT	FGF7 + IWP2						
	Stage 3: PP1	FGF7 + RA + TPB + LDN + SANT1						
	Stage 4: PP2	FGF7 + RA + TPB + LDN + SANT1						
	Stage 5: EP	RA + SANT1 + ALK5iII + T3 + LDN						
	Stage 6: β cell	ALK5iII + T3 + LDN + XXI						
(Hogrebe et al., 2020)	Stage 1: DE	Activin A + CHIR	HUES8	Planar culture	Stage 6/ No	n.d	~ 80% CHGA^+^; ~ 54% NKX6-1^+^/CHGA^+^	~ 40% C-peptide^+^/ NKX6-1^+^
	Stage 2: PGT	KGF						
	Stage 3: PP1	KGF + RA + TPPB + LDN + SANT1						
	Stage 4: PP2	KGF + RA + TPPB + LDN + SANT1						
	Stage 5: EP	RA + SANT1 + ALK5iII + T3 + XXI + Betacellulin + Latrunculin A						
	Stage 6: β cell	ESFM						
(Yoshihara et al., 2020)	Stage 1: DE	Activin A + CHIR	HUES8	Suspension culture	No/ No	n.d	n.d	50% ~ 60% insulin^+^/ NKX6-1^+^
	Stage 2: PGT	FGF7						
	Stage 3: PP1	FGF7 + RA + TPB + LDN + SANT1 + ALK5iII						
	Stage 4: PP2	FGF7 + RA + SANT1 + LDN + ALK5iII						
	Stage 5: EP	SANT1 + ALK5iII + T3 + LDN + GSiXX						
	Stage 6: β cell	ALK5iII + T3 + N-Cys + R428 + rhWNT4						
(Liu et al., 2021)	Stage 1: DE	Activin A (115–111-100 ng/ml) + CHIR	H1	Planar culture/air–liquid interface	Stage 5/ No	81 ± 4% PDX1^+^/NKX6-1^+^	n.d	60% ~ 82% insulin^+^/ NKX6-1^+^
	Stage 2: PGT	KGF + Dorsomorphin						
	Stage 3: PP1	KGF + RA + Noggin + SANT1						
	Stage 4: PP2	EGF + Nicotinamide + Noggin						
	Stage 5: PP-10C	LDN + T3 + SANT1 + Repsox + RA + ZnSO_4_ + TPB + EGF + Nicotinamide + GABA						
	Stage 6: EP	FSK + LDN + TBP + Repsox + KGF + SANT1 + RA + T3						
	Stage 7: IB	LDN + T3 + Repsox + ZnSO4 + GSiXX + RA + HGF + IGFI + PD						
	Stage 8: β cell	BTC + ISX-9 + G-1 + Deza + ZM447439 + H1152 + CI-1033						

*DE* Definitive Endoderm, *PGT* Primitive Gut Tube, *PP1* Pancreatic Progenitors 1, *PP2* Pancreatic Progenitors 2, *EP* Endocrine Precursors, *IB* Immature β cells, *PP-10C* Pancreatic Progenitors treated with 10 compounds, *ND* Non –diabetic, *T1D* Type 1 diabetic, *n.d*. *N*ot determined

The signaling pathways manipulated in differentiation require further investigation as well. As an example, at the final β cell maturation stage, inhibition of the TGF-β signaling pathway by ALK5iII has been applied to obtain mature and functional pancreatic β cells (Pagliuca et al., 2014; Rezania et al., 2014; Ghazizadeh et al., 2017), whereas Velazco-Cruz and colleagues recently demonstrated that allowing the TGF-β signaling pathway was indispensable for robust dynamic function of β cells (Velazco-Cruz et al., 2019). Russ and colleagues found that the use of BMP inhibitor to designate pancreatic progenitor cells resulted in precocious induction of endocrine differentiation leading to the formation of polyhormonal cells (Russ et al., 2015), which is not consistent with other in vitro differentiation protocols (Pagliuca et al., 2014; Rezania et al., 2014). Liu and colleagues identified several new chemicals useful for pancreatic β cell differentiation, including forskolin (cAMP pathway activator) for the induction of endocrine progenitors, ISX-9 (Neurod1 inducer), G-1 (G protein-coupled estrogen receptor agonist), Deazaneplanocin A (histone methyltransferase inhibitor), ZM447439 (aurora kinase inhibitor), and CI-1033 (pan-ErbB inhibitor) for the maturation of β cells; however, the molecular mechanism of those chemicals or signals remains largely unrevealed (Liu et al., 2021). Moreover, the pancreas is derived from both the dorsal and ventral endoderm, regulated by different signals and regulatory factors, which indicates distinct pancreatic specification regulatory mechanisms from these two regions (Li et al., 2018; Li et al., 2021; Larsen and Grapin-Botton, 2017). The pancreatic progenitors currently differentiated from human PSCs in vitro seem to be closer to dorsal than ventral pancreatic buds, indicating that stepwise pancreatic differentiation follows dorsal pancreatic program in vitro (Jennings et al., 2017). The extent to which differences in dorsal and ventral pancreatic program affect in vitro differentiation of β cells is also unclear.

It is obvious that the cell cultures become highly heterogeneous as PSC-derived β cell differentiation progresses. In particular, the induction of endocrine cells involves a diverse population of endocrine cells, undifferentiated progenitor cells and even exocrine cells, and ultimately only a small population of insulin-expressing β cells (Petersen et al., 2017; Weng et al., 2020). Surprisingly, the enterochromaffin cells, which synthesize and secrete serotonin in the gut in nature, were found during in vitro pancreatic β cell differentiation (Veres et al., 2019). The presence of ZM447439 in the final maturation stage decreased the proportion of the enterochromaffin cells (Balboa et al., 2022). Removing unwanted cells from the final product is critical for clinical transplantation. Single-cell RNA sequencing is a promising technique to study complex tissues and organs and determine the developmental mapping of cell lineage. It has been used to describe cell heterogeneity during differentiation and define molecular regulatory mechanisms of pancreatic lineage development, rapidly advancing the understanding of β cell fate determination and functional maturation (Veres et al., 2019; Chen et al., 2019; Yu and Xu, 2020). Furthermore, epigenome analysis of DNA methylation, chromatin accessibility, and histone modification complements the regulatory mechanisms from an epigenetic perspective (Gaertner et al., 2019; Alvarez-Dominguez et al., 2020).

Glucose responsiveness is the key to pancreatic β cell functional maturation. Although human PSC-derived β cells can undergo the dynamic GSIS with first- and second-phase insulin secretion, the amount of insulin secreted in the second stage is low (Velazco-Cruz et al., 2019; Nair et al., 2019; Veres et al., 2019) (Table 2), indicating that the resulting β cells are still less functional than cadaveric islets. In addition, functional β cells are characterized by an increased rate of mitochondrial oxidative phosphorylation. It was found that the insufficient ability of PSC-derived β cells to secrete insulin in response to glucose was due to metabolic failure caused by reduced anaplerotic cycling in the mitochondria (Davis et al., 2020). The pancreatic β cells differentiated from human PSCs exhibit immature mitochondrial glucose coupling (Balboa et al., 2022). Achieving metabolic maturation of pancreatic β cells contributes to their functional maturation. Moreover, the microenvironment and the structure of the islet need to be taken into account in order to mimic the function of primary pancreatic β cells more accurately. Dynamic GSIS indicating the in vitro maturation of β cell differentiation was found to be associated with the cytoarchitectural reorganization and the increasing presence of α cells (Balboa et al., 2022). Apart from endocrine cells, the islet also contains endothelial cells, pericytes, resident immune cells and so on (Almaca et al., 2018; Walker et al., 2021). Consequently, co-culturing β cells with other endocrine types, endothelial cells, and immune cells and providing vascularized networks could improve functional maturation (Zhang et al., 2021; Siehler et al., 2021; Cozzitorto et al., 2020) (Fig. 3).

**Table 2:** Function assessment of human PSC-derived β cells in Table 1

Work	In vitro function			In vivo function		
	**In vitro insulin secretion**	**Static GSIS**	**Dynamic GSIS**	**Number of β cell transplanted**	**Stimulation index** **(Earliest time point)**	**Diabetes reversal (Earliest time point)**
(Rezania et al., 2014)	n.d	1.4–3.3 (CP)	No	1.25 × 10^6^ cells	~ 1.4 (CP) (2 weeks)	Yes (40 days)
(Pagliuca et al., 2014)	1.6 ± 0.2 μIU/10^3^ cells	2.2 ± 0.3 (INS)	No	5 × 10^6^ cells	1.7 ± 0.2 (INS) (2 weeks)	Yes (18 days)
(Russ et al., 2015)	2.5 ± 1.2 μg/ug DNA	1.8 ± 0.9 (CP)	No	1.15 × 10^6^ cells	~ 1.3 (INS) (7 ~ 10 days)	Lack of complete diabetes reversal
(Millman et al., 2016)	(ND) 1.9 ± 0.3 μIU/10^3^ cells; (T1D) 2.0 ± 0.4 μIU/10^3^ cells	(ND) 2.2 (INS); (T1D) 1.9 (INS)	No	5 × 10^6^ cells	(ND) 1.5 ± 0.2 (INS) (4 weeks); (T1D) 1.4 ± 0.3 (INS) (4 weeks)	Yes (Not mentioned)
(Ghazizadeh et al., 2017)	n.d	~ 3 (CP)	No	2 × 10^6^ cells	< 2 (INS) (5 weeks)	n.d
(Velazco-Cruz et al., 2019)	5.3 ± 0.5 μIU/10^3^ cells	3.0 ± 0.1 (INS)	First phase Stimulation: 7.6 ± 1.3 (INS); Second phase Stimulation: 2.1 ± 0.3 (INS)	5 × 10^6^ cells	~ 2 (INS) (10 weeks)	Yes (Not mentioned)
(Veres et al., 2019)	n.d	~ 3.4 (INS)	First phase Stimulation: 3.21 (INS); Second phase Stimulation: ~ 1.5 (INS)	n.d		
(Rosado-Olivieri et al., 2019)	n.d	~ 3 (INS)	No	5 × 10^6^ cells	~ 1.7 (INS) (8 weeks)	n.d
(Nair et al., 2019)	n.d		First phase Stimulation: ~ 4 (CP); Second phase Stimulation: ~ 1 (CP)	4 × 10^6^ cells	~ 5 (CP) (8 months)	n.d
(Mahaddalkar et al., 2020)	n.d	~ 2.2 (INS)	First phase Stimulation: ~ 5 (INS); Second phase Stimulation: ~ 1.15 (INS)	n.d		
(Hogrebe et al., 2020)	n.d		First phase Stimulation: 9.43 (INS); Second phase Stimulation: 1.88 (INS)	5 × 10^6^ cells	~ 3 (INS) (2 weeks)	Yes (2 weeks)
(Yoshihara et al., 2020)	n.d	~ 3.5 (CP)	No	n.d	n.d	Yes (Not mentioned.)
(Liu et al., 2021)	~ 62 ng/10^3^ cells	~ 2.7 (CP)	No	n.d	n.d	Yes (2 weeks)

*INS* Insulin, *CP* C-peptide, GSIS represented as stimulation index. *ND* Non –diabetic, *T1D* Type 1 diabetes, n.d. *N*ot determined

The clinical translation of PSC-based islet replacement strategy for diabetes mellitus remains challenging. Mass production of PSC-derived β cells with high purity is essential for transplantation. Three-dimensional culture is conducive to large-scale manufacturing, while optimization of differentiation protocol and purification and enrichment with β cell-specific markers contribute to the removal of unwanted cells. Importantly, long-term survival and function of the grafts are crucial to PSC-based islet replacement therapy. The death of grafted cells after transplantation not only reduces therapeutic efficiency but also stimulates immune attack. Apart from improving the encapsulation strategy and generating immune-evasive cells for transplantation, the transplantation site also matters for this concern. The kidney capsule has been the most commonly used transplantation site in mouse models, but the clinical translation ability is limited. Most of clinical human islet transplantations have been performed at the hepatic portal vein. This operation is minimally invasive, and enables transplanted β cells to physiologically release insulin as well as provides oxygenation to the transplanted islets via the portal circulation. But the hepatic microenvironment is not ideal as severe early cell loss happens due to instant blood-mediated inflammatory reaction (IBMIR) and hypoxic apoptosis. Subcutaneous transplantation is easy to operate with minimally invasive delivery and few surgical complications and is accessible to monitor and retrieve the grafts, but there is low vascular density. Other more vascularized and/or immune-privileged sites also have limitations; for instance, the omentum needs an invasive operation, and the anterior chamber of the eye is only suitable for blind patients (Cayabyab et al., 2021). Ideally, transplanting unencapsulated or minimally physiologically impaired, robustly functional human PSC-derived β cells without compromising the immune system of the recipient is optimal for the PSC-based islet replacement therapy, which needs constant effort.

In conclusion, pancreatic β cells with certain functions can be derived efficiently from human PSCs in vitro stepwise differentiation and alleviate hyperglycemia after transplantation in animal models. Critically, to eventually obtain β cells more comparable to human primary β cells, the mechanism by which immature β cells are directed into functional mature β cells needs to be further studied. The constant efforts will contribute to the pathogenesis and therapies of diabetes mellitus and improve the biological knowledge of human pancreatic β cells.

## Data Availability

Not applicable.

## Abbreviations

MODY: Maturity-onset diabetes of the young
ESC: Embryonic stem cell
iPSC: Induced pluripotent stem cell
PSC: Pluripotent stem cell
EPSC: Extended pluripotent stem cell
dpc: Days post conception
TGF-β: Transforming growth factor-β
WNT: Wingless and Int-1
GSK3-β: Glycogen synthase kinase 3-β
BMP: Bone morphogenetic protein
FGF: Fibroblast growth factor
PI3K: Phosphoinositide 3 kinase
JNK: Jun N-terminal kinase
KGF: Keratinocyte growth factor
SHH: Sonic hedgehog
RA: Retinoic acid
PKC: Protein kinase C
EGF: Epidermal growth factor
wpc: Weeks post conception
GSIXX: Gamma-secretase inhibitor XX
GSIS: Glucose-stimulated insulin secretion
HLA: Human leukocyte antigen
PD-L1: Programmed death ligand-1
IBMIR: Instant blood-mediated inflammatory reaction
DE: Definitive Endoderm
PGT: Primitive Gut Tube
PF: Posterior Foregut
PE: Pancreatic Endoderm
EP: Endocrine Precursor
