# Correction: Stepwise differentiation of functional pancreatic β cells from human pluripotent stem cells

**DOI:** 10.1186/s13619-022-00134-7

**Published:** 2022-08-23

**Authors:** Wenwen Jin, Wei Jiang

**Affiliations:** 1 Department of Biological Repositories, Frontier Science Center for Immunology and Metabolism, Medical Research Institute, Zhongnan Hospital of Wuhan University, Wuhan, 430071 China; 2 Human Genetics Resource Preservation Center of Wuhan University, Wuhan, 430071 China

**Correction: Cell Regeneration 11, 24 (2022)**

**https://doi.org/10.1186/s13619-022-00125-8**

Following publication of the original article (Jin & Jiang, 2022), it was reported that the word “financial” needs to be removed from the Competing interests section.

The original sentence was:

The authors declare no competing financial interests.

The correct sentence should be:

The authors declare no competing interests.

The original article (Jin & Jiang, 2022) has been corrected.
